# Validation of New Instrumentation for Isotope Dilution Mass Spectrometric Determination of Organic Serum Analytes

**DOI:** 10.6028/jres.104.010

**Published:** 1999-04-01

**Authors:** P. Ellerbe, C. S. Phinney, L. T. Sniegoski, M. J. Welch

**Affiliations:** National Institute of Standards and Technology, Gaithersburg, MD 20899-0001

**Keywords:** definitive methods, isotope dilution mass spectrometry, measurement

## Abstract

A major activity in the 20 year collaboration between the Analytical Chemistry Division at NIST and the College of American Pathologists (CAP) has been the development of highly accurate and precise “definitive” methods for important clinical analytes in human serum. Definitive methods for organic analytes use isotope dilution/gas chromatography/mass spectrometry and require a mass spectrometer capable of making highly precise measurements of the ratio between the ion intensities of a characteristic ion from the analyte of interest and its stable-isotope-labeled analog. Recently, the mass spectrometer used for 20 years for definitive method development and measurements was replaced with a modern instrument capable of automated operation, with accompanying gains in convenience and sample throughput. Switching to the new instrument required modifications of measurement protocols, acceptance criteria, and ratio calculations with background corrections to go along with automated instrument operation. Results demonstrated that the two instruments gave comparable results for measurements of both urea and cholesterol in samples from various serum-based Standard Reference Materials [SRMs] and College of American Pathologists materials.

## 1. Introduction

As part of a program for standardization of clinical methods, the Analytical Chemistry Division at the National Institute of Standards and Technology, in cooperation with the College of American Pathologists (CAP), has undertaken the development of definitive methods for several clinically important analytes in human serum: cholesterol [1, 2], glucose [3], uric acid [4, 5], urea [6], creatinine [7], and triglycerides [8].

The National Committee for Clinical Laboratory Standards has published guidelines for definitive methods [9], which define a set of rules for the acceptance or rejection of a given method as definitive.

Isotope dilution mass spectrometry (ID/MS) is the technique of choice for definitive methods for most common clinical analytes, since it does not depend on sample recovery, generates results with high precision, and can be tested for bias and interferences for which the identity of the interfering compound is unknown.

The use of ID/MS for organic analytes is based on adding a known amount of an isotopically labeled version of the analyte to the sample as an internal standard, equilibrating the labeled analyte with the endogenous analyte, processing the sample, and then measuring the ratio of unlabeled-to-labeled analyte by using gas chromatography-mass spectrometry (GC/MS). Assuming complete equilibration with the labeled analyte after spiking, less than complete recovery of the analyte does not affect the measured concentrations, unless there is a significant isotope effect, since it is the ratio of unlabeled to labeled analyte that is measured. (Generally, deuterium is the only stable isotope used in labeling organic compounds for which significant isotope effects can be seen.) Random variation in sample preparation is evaluated by preparing independent multiple sets of samples.

Although the probability of a significant measurement interference is low when a compound class isolation procedure followed by capillary column GC/MS is used, it still would be possible for a substance to coelute from the GC with the measured species, contribute to the ion intensity measured for either the unlabeled or labeled form, and thus interfere with the measurement. This probability of undetected interferences is even further reduced by measuring all samples with the use of a prominent ion from electron impact ionization, and then selecting a representative subset of samples that are measured at other prominent ions, with other GC columns, and/or with another mode of ionization. Therefore, for an interfering species to be undetected, it would have to have the same retention time as that of the analyte on the different GC columns, the same ions at all masses used for measurement in each mode of ionization, and the same abundance ratios among these ions as that of either the unlabeled or labeled version of the analyte being measured. Such a situation is highly unlikely. Even interferences of which the nature is unknown can be detected by this approach.

The mass spectrometer used for definitive methods must be capable of measuring intensity ratios to the precision required (typically coefficients of variation of < 0.5 %). For more than 20 years we have used a Varian CH7 mass spectrometer^2^ that has been extensively modified to achieve this purpose [3]. In 1997 a JEOL MStation-700 instrument was acquired for use in these measurements. Because the definitive methods are described in explicit detail, any changes in the methods require documentation to demonstrate that the modifications do not adversely affect the quality of the results. In this paper we describe the new instrument, discuss the changes in the measurement protocol and the calculation program that were made, and demonstrate that the two instruments give comparable results for two of the analytes for which definitive methods are available: urea and cholesterol.

## 2. Experimental Details

### 2.1 Materials

Samples for urea measurements were selected from a group of sets previously prepared for the analyses of Standard Reference Materials [SRMs] 909a and 909b (Human Serum) and selected CAP Survey materials. Samples for cholesterol measurements were selected from a group of sets previously prepared for the analysis of SRM 1951a (Lipids in Frozen Human Serum) and SRM 909b.

### 2.2 Sample Preparation

The sample preparation for cholesterol and urea has been previously described [2, 6]. The cholesterol derivative is the trimethylsilyl ether; the urea derivative is 6-methyl-2-4-bis[(trimethylsilyl)-oxy]pyrimidine. The derivatized cholesterol samples were measured using the CH7 in 1996 and the MStation-700 in 1997; the derivatized urea samples were measured using the CH7 in 1995 and the MStation-700 in 1997.

### 2.3 Instrumentation

The Varian CH7 instrument and the GC attached to it (Varian Instruments, Palo Alto, CA), as well as the extensive modifications made to the system, have been previously described [1, 2, 5].

The MStation-700 (JEOL, Peabody, MA) is a fully automated computer-controlled mass spectrometer system. Its ion optics were designed by Matsuda [10] for high ion beam acceptance and are of the reverse-geometry type. An accelerating voltage of up to 10 kV can be used. The ion detection system is a discrete dynode electron multiplier with a conversion dynode on the front end.

The GC is a Hewlett-Packard Model 6890, equipped with a computer-controlled autosampler. The computer systems of the GC and the mass spectrometer are networked together.

### 2.4 GC/MS Conditions

The GC/MS conditions used with the CH7 in the measurement of cholesterol and urea have been previously described [2, 6].

The GC/MS conditions used with the MStation-700 in the measurement of urea were as follows. For the MS, the accelerating voltage was set at 8 kV and the slits were set to obtain a resolution of at least 1000. The filament was turned on 5 min after injection. The multiplier was at 1.0 kV, the source temperature and transfer lines were at 200 °C. The ionizing current was 100 mA, which resulted in an emission current of about 250 mA at an ionizing voltage of 70 eV. The exact mass to charge ratios (*m/z*) used were 255.0985 and 257.1028. The GC column used was a 30 m long, 0.25 mm inside diameter nonpolar proprietary phase fused silica capillary column with a 0.25 m film thickness which is designed specially for use with MS (DB-5-ms, J&W Associates, Folsom, CA). The column was run under constant flow conditions at 0.6 mL/min He. The mode of injection was splitless, with an injector temperature of 150 °C. The column temperature was 70 °C initially for 1 min, followed by a rise at 40 °C per min to 140 °C. The retention time of urea under these conditions is about 7.9 min.

The GC/MS conditions used with the MStation-700 in the measurement of cholesterol were as follows. For the MS, the accelerating voltage was set at 8 kV and the slits were set to obtain a resolution of at least 1000. The filament was turned on 3 min after injection. The multiplier was at 0.8 kV, the source temperature and transfer lines were at 200 °C. The ionizing current was 300 mA, which resulted in an emission current of about 900 μA at an ionizing voltage of 70 eV. The exact m/z values were 458.3944 and 461.4045. The GC column used was the same as that used for the urea measurements. The column was run under constant flow conditions at 0.6 mL/min He. The mode of injection was split, with a split ratio of 20:1 and with an injector temperature of 300 °C. The column temperature was 295 °C (isothermal). The retention time of cholesterol under these conditions is about 6.3 min.

### 2.5 Calculation Program

The program used to calculate the ion intensity ratios when using the CH7 has been previously described [1, 2].

The program used to calculate the ion intensity ratios when using the MStation-700 is as follows. The peak maximum for the unlabeled mass is identified. The threshold that has been selected by the operator (4 % in this case) is used to determine when to start and stop the integration. The first cycle before the threshold is reached on the front of the peak and the first cycle below the threshold on the back of the peak are included. The counts are summed along this segment. The number of cycles included is counted. The baseline is found by going back the number of seconds specified by the operator before the peak maximum and summing the same number of cycles, up to that point, as are used for the integration. This sum is subtracted from the integration sum. The exact same cycles are used for the labeled mass integration calculations. Finally, the unlabeled baseline-corrected integration sum is divided by the labeled baseline-corrected integration sum to give the ion intensity ratio.

## 3. Results and Discussion

### 3.1 The Reason for Changing Instrumentation

The CH7 was still functional, but because it was nearly 20 years old, the downtime necessary for repairs was increasing and replacement parts were becoming unavailable. In addition, the CH7 could not be automated, thereby limiting the throughput.

The MStation-700 is supported completely by JEOL, and was automated, with the increase in sample throughput that automation brings.

### 3.2 Measurement Protocol

The measurement protocol used with the CH7 has been described earlier [1]. This protocol requires the attendance of an operator to make the necessary decisions about injection order.

The MStation-700 is normally used unattended with the autosampler in operation. Therefore, some changes in the measurement protocol were made. In the previous protocol, each standard and sample were measured twice in succession, and these intensity ratios were acceptable only if they agreed within 0.5 % of each other. If either of these criteria were not met, then a third measurement was made, whose intensity ratio had to agree within 0.5 % of one of the other two measurements, and then all three were averaged. (In the rare occasion that the third intensity ratio did not agree with one of the other two measurements, all three were discarded, and the measurement repeated.) Also, each time a standard was used again in any given half day, only a single intensity ratio was obtained, as long as the new intensity ratio was within 0.5 % of the previous value for that standard. In addition, for each standard-sample-standard group the calculated peak areas had to be within a factor of two of each other, or the measurement was discarded. These parts of the protocol require that the operator be present to make a decision about the identity of the next injection.

The new protocol requires that each standard and sample be injected twice. After the data are collected, the intensity ratios and calculated peak areas are examined by the operator. The duplicate intensity ratios must be within 0.5 % of each other for each sample and standard to be acceptable. If the duplicates do not agree, the measurements affected by the disagreement are discarded, and those samples are remeasured. In addition, for each standard-sample-standard group the calculated peak areas must be within a factor of four of each other, or the measurement is discarded.

In the old protocol, measurements were made of each sample on two separate days; the definition of the word “day” is obvious when an operator is involved. The MStation-700 however can run all day and all night, so that the meaning of the word “day” is no longer clear; therefore, the “different day” concept has been abandoned. In performing a single-ion-monitoring (SIM) experiment, the MStation-700 puts all the runs into one file. Only after the file is closed can data analysis and calculations begin. Now the protocol simply requires that the two measurements be in different SIM files. This also means that the two measurements have been performed using two different peak adjustment files, since each SIM file has its own peak adjustment file, and it therefore also means that the two measurements have been performed under at least slightly different conditions, since the instrument parameters change at least slightly in each peak adjustment file.

### 3.3 Selection of Analytes for Measurement

The analytes chosen for this comparison had to be stable as derivatives for several years, and samples that had been measured on the CH7 had to be still available. Cholesterol and urea fit these requirements. Cholesterol is also the analyte that is most often the subject of these high-precision measurements.

### 3.4 Results

When the various materials were measured for each analyte, the results obtained from the different mass spectrometers were in excellent agreement (Table 1). The bias between the instruments for urea ranged from −0.12 % to −0.39 % on the same samples using the CH7 and the Mstation-700. For urea, the biases were in only one direction, but even the largest one was only −0.39 %. This bias may arise from changes in the samples during the two years between measurements by the two instruments.

The bias between the instruments for cholesterol ranged from −0.15 % to +0.17 %. The biases are in both directions. Samples prepared from both frozen and freeze-dried serum were tested, and the results were equally good.

Precision is also an important attribute of the definitive methods. For those cases where sufficient samples were measured from a given material, the sample-sample precision was compared. The coefficients of variation were very similar with the two instruments. The precision obtained using the MStation-700 was marginally better than that obtained using the CH7 in five cases and marginally worse in the other two cases; thus the two instruments produce results of similar precision. This comparison demonstrates that with the MStation-700, it is possible to obtain high precision without the need for the presence of the operator, which was required with the CH7.

## 4. Conclusions

The agreement between values obtained on the original instrument CH7 and on the new instrument MStation-700 is excellent. Therefore, we have demonstrated that the two instruments provide comparable results with comparable precisions for two of the analytes for which definitive methods have been developed.

## Figures and Tables

**Table 1:** Comparison of concentrations obtained in samples (mmol/L), summarized by material measured

Material	Number of samples	MStation–700 value [CV(%)]	CH7 value [CV(%)]	Difference^a^
Urea				

SRM 909a^b^	2	19.443	19.470	−0.14 %
level 2		(0.12 %)	(0.16 %)	
SRM 909b^b^	2	5.468	5.476	−0.15 %
level 1		(0.04 %)	(0.35 %)	
SRM 909b^b^	5	30.635	30.732	−0.31 %
level 2		(0.28 %)	(0.24 %)	
CAP C–02^b^	1	8.142	8.165	−0.29 %
1996				
CAP C–10^b^	1	16.377	16.397	−0.12 %
1996				
CAP 1996^c^	1	4.232	4.249	−0.39 %
Frozen 1				

Cholesterol				

SRM 1951a^c^	9	4.704	4.711	−0.15 %
level 1		(0.18 %)	(0.28 %)	
SRM 1951a^c^	9	7.168	7.156	+0.17 %
level 2		(0.26 %)	(0.21 %)	
SRM 909b^b^	3	3.791	3.784	+0.18 %
level 1		(0.06 %)	(0.09 %)	
SRM 909b^b^	3	6.052	6.056	−0.07 %
level 2		(0.38 %)	(0.45 %)	

a Difference = ([MStation–700 Concentration–CH7 Concentration] / CH7 Concentration).

b Freeze-dried serum.

c Frozen serum.

## Footnotes

2 Certain commercial equipment, instruments, or materials are identified in this paper to foster understanding. Such identification does not imply recommendation or endorsement by the National Institute of Standards and Technology, not does it imply that the materials or equipment identified are necessarily the best available for the purpose.
